# Automated lesion detection of breast cancer in [^18^F] FDG PET/CT using a novel AI-Based workflow

**DOI:** 10.3389/fonc.2022.1007874

**Published:** 2022-11-15

**Authors:** Jeffrey P. Leal, Steven P. Rowe, Vered Stearns, Roisin M. Connolly, Christos Vaklavas, Minetta C. Liu, Anna Maria Storniolo, Richard L. Wahl, Martin G. Pomper, Lilja B. Solnes

**Affiliations:** ^1^ The Russell H. Morgan Department of Radiology and Radiological Science, Johns Hopkins University School of Medicine, Baltimore, MD, United States; ^2^ Department of Oncology, Sidney Kimmel Comprehensive Cancer Center, Johns Hopkins University School of Medicine, Baltimore, MD, United States; ^3^ Cancer Research @ UCC, College of Medicine and Health, University College Cork, Cork, Ireland; ^4^ Huntsville Cancer Institute, University of Alabama, Birmingham, AL, United States; ^5^ Division of Medical Oncology, Mayo Clinic, Rochester, MN, United States; ^6^ Melvin and Bren Simon Cancer Center, Indiana University, Indianapolis, IN, United States; ^7^ Mallinckrodt Institute of Radiology, Washington University School of Medicine, St. Louis, MO, United States

**Keywords:** artificial intelligence, machine learning, deep learning, PERCIST v1.0, breast cancer

## Abstract

Applications based on artificial intelligence (AI) and deep learning (DL) are rapidly being developed to assist in the detection and characterization of lesions on medical images. In this study, we developed and examined an image-processing workflow that incorporates both traditional image processing with AI technology and utilizes a standards-based approach for disease identification and quantitation to segment and classify tissue within a whole-body [^18^F]FDG PET/CT study.

**Methods:**

One hundred thirty baseline PET/CT studies from two multi-institutional preoperative clinical trials in early-stage breast cancer were semi-automatically segmented using techniques based on PERCIST v1.0 thresholds and the individual segmentations classified as to tissue type by an experienced nuclear medicine physician. These classifications were then used to train a convolutional neural network (CNN) to automatically accomplish the same tasks.

**Results:**

Our CNN-based workflow demonstrated Sensitivity at detecting disease (either primary lesion or lymphadenopathy) of 0.96 (95% CI [0.9, 1.0], 99% CI [0.87,1.00]), Specificity of 1.00 (95% CI [1.0,1.0], 99% CI [1.0,1.0]), DICE score of 0.94 (95% CI [0.89, 0.99], 99% CI [0.86, 1.00]), and Jaccard score of 0.89 (95% CI [0.80, 0.98], 99% CI [0.74, 1.00]).

**Conclusion:**

This pilot work has demonstrated the ability of AI-based workflow using DL-CNNs to specifically identify breast cancer tissue as determined by [^18^F]FDG avidity in a PET/CT study. The high sensitivity and specificity of the network supports the idea that AI can be trained to recognize specific tissue signatures, both normal and disease, in molecular imaging studies using radiopharmaceuticals. Future work will explore the applicability of these techniques to other disease types and alternative radiotracers, as well as explore the accuracy of fully automated and quantitative detection and response assessment.

## Introduction

The application of deep learning (DL)-based artificial intelligence (AI) as a tool for automated interpretation of radiologic images is a fast-growing area of investigation (1, 2). However, many of these efforts have been focused on the application of AI to identify structural elements (either normal or abnormal anatomy) in computed tomography (CT) or magnetic resonance imaging (MRI) (3–7). Within the field of molecular imaging, the application of AI for advancing image acquisition and reconstruction (8–10), attenuation correction methods (8, 11), and lesion identification (8, 12, 13) are areas of active research.

Here we are expanding upon our earlier work (12) to investigate how DL-based AI may enhance automatic segmentation and classification of tissue based on location and avidity levels in 2-deoxy-2-[^18^F]fluoro-D-glucose ([^18^F]FDG) positron emission tomography (PET)/CT studies, with a specific focus on the detection of breast cancer.

Our approach utilizes a convolutional neural network (CNN) as the underlying DL engine. CNNs consist of an input layer (the source images, such as our PET/CT images), an output layer (a pixel classification map), and one or more ‘hidden’ layers connecting the two. Hidden layers are composed of interconnected ‘perceptrons’ (algorithms that decide if an input, or inputs, belong to a specific class) that provide connectivity between the different nodes in the network, essentially constructing a very high number of computational paths between an input image pixel and the output classifier pixel. They are called ‘convolutional’ networks when a convolutional operation is performed in at least one or more of the hidden layers. The network can be trained to classify specific pixels in an image if provided with enough input images that are paired with ‘ground truth’ classification data by iteratively adjusting the weights used by each perceptron until the system achieves a pre-determined level of accuracy predicting the provided ‘ground truth’ classifications.

**Figure 1:**
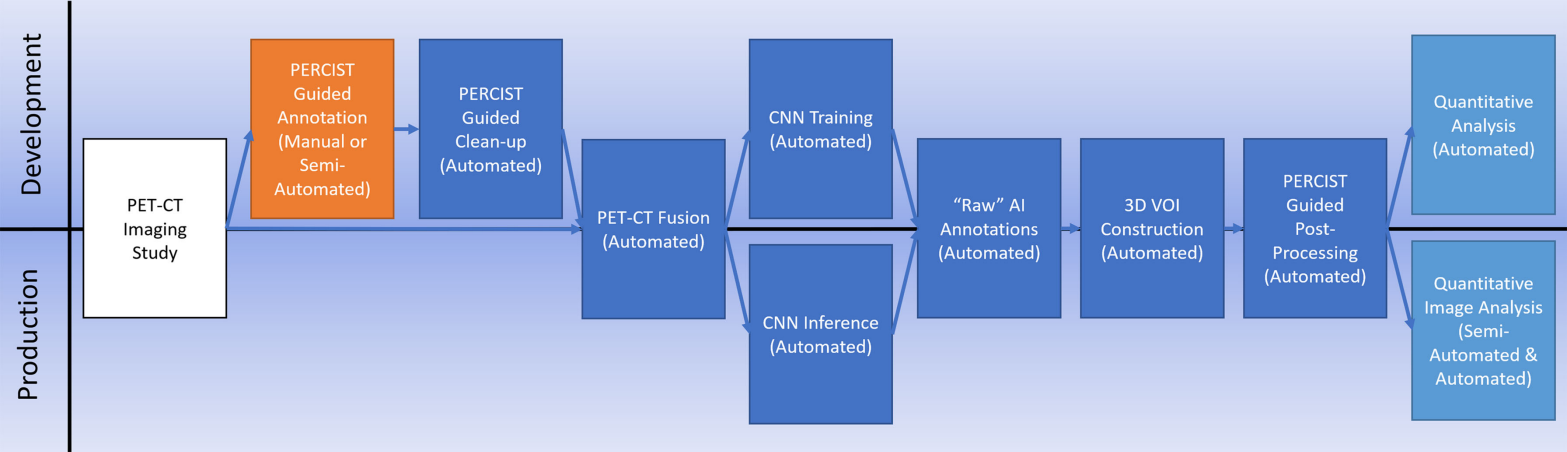
Our AI processing workflow illustrating both training and production pipelines.

It is our hypothesis that a workflow (**Figure 1**) that synthesizes existing standards-based techniques for data processing with an appropriately architected CNN can result in an AI system capable of clinical-level performance. We tested this hypothesis on breast cancer for this initial study based on the availability of a diverse collection of prospectively acquired imaging from two separate multi-institutional clinical trials.

## Methods

### PET imaging studies

For this study we used baseline [^18^F]FDG PET/CT imaging exams acquired as part of two separate multi-institutional clinical trials of patients with breast cancer. The first was TBCRC 008 (NCT00616967) (14), which examined the rate of complete response when using chemotherapy vs chemotherapy + vorinostat in cases of HER2-negative breast cancer. The second was TBCRC 026 (NCT01937117) (15), a trial that examined early changes in PET standardized uptake values corrected for lean body mass (SUL) as predictors of pathologic complete response in cases of HER2-positive breast cancer when treated with pertuzumab and trastuzumab. Although representing different clinical patient cohorts, this study only used the baseline PET studies, which were similar radiological cohorts. The patient characteristics for each study were similar [**Table 1**]. Only those patients from each study for which their PET/CT scans met technical requirements of uniformity in acquisition and who were able to undergo both the baseline and follow-up imaging were included in our study, resulting in a total of 130 cases total.

**Table 1:** Demographic and disease characteristics of patients included in this study.

Characteristic	TBCRC008	TBCRC026	Total
No. of Patients	60 (of 62)	70 (of 88)	130 (of 150)
Age (y)			
Median	48	58	53
Range	24-72	29-82	24-82
Race			
Caucasian	43	75	118
Black	13	7	20
Other	6	6	12
ECOG			
0	59	76	135
1	3	12	15
Tumor Size, cm			
Median	4	3.7	3.8
Range	1.5-18	2.0-15	1.5-18
Tumor Grade			
2	18	22	40
3	44	66	110

A notable characteristic of this dataset was its multi-institutional sourcing. Images were obtained from 11 different institutions and were acquired on 8 different PET/CT scanners from 3 different manufacturers.

### Software and hardware environment

We used an HP G4 Z4 workstation equipped with a Zeon processor, 128 GB RAM, and an Nvidia A6000 GPU. For the neural network we used MATLAB (16) as well as in-house developed software (17, 18) using Java.

### Training data preparation

Preparation of the PET/CT image data and corresponding voxel classification maps was a multi-step process. The first step involved the generation of the initial training annotations and for this we used our in-house developed Auto-PERCIST (17) software. This software automates the mechanics of PERCIST v1.0 (19) analysis by transforming PET data into SUL and then calculating a global PERCIST baseline assessment threshold based on an automated measurement sampled from the liver for use as a global segmentation threshold. PERCIST v1.0 defines this baseline assessment threshold (19) as:


$$\text{Baseline Assessment Threshold}=1.5\times {\text{Mean}}_{\text{Liver}}+2.0\times \text{Std}.\text{Dev}._{\text{Liver}}$$

  Equation 1 Baseline Assessment Threshold

**Figure 2:**
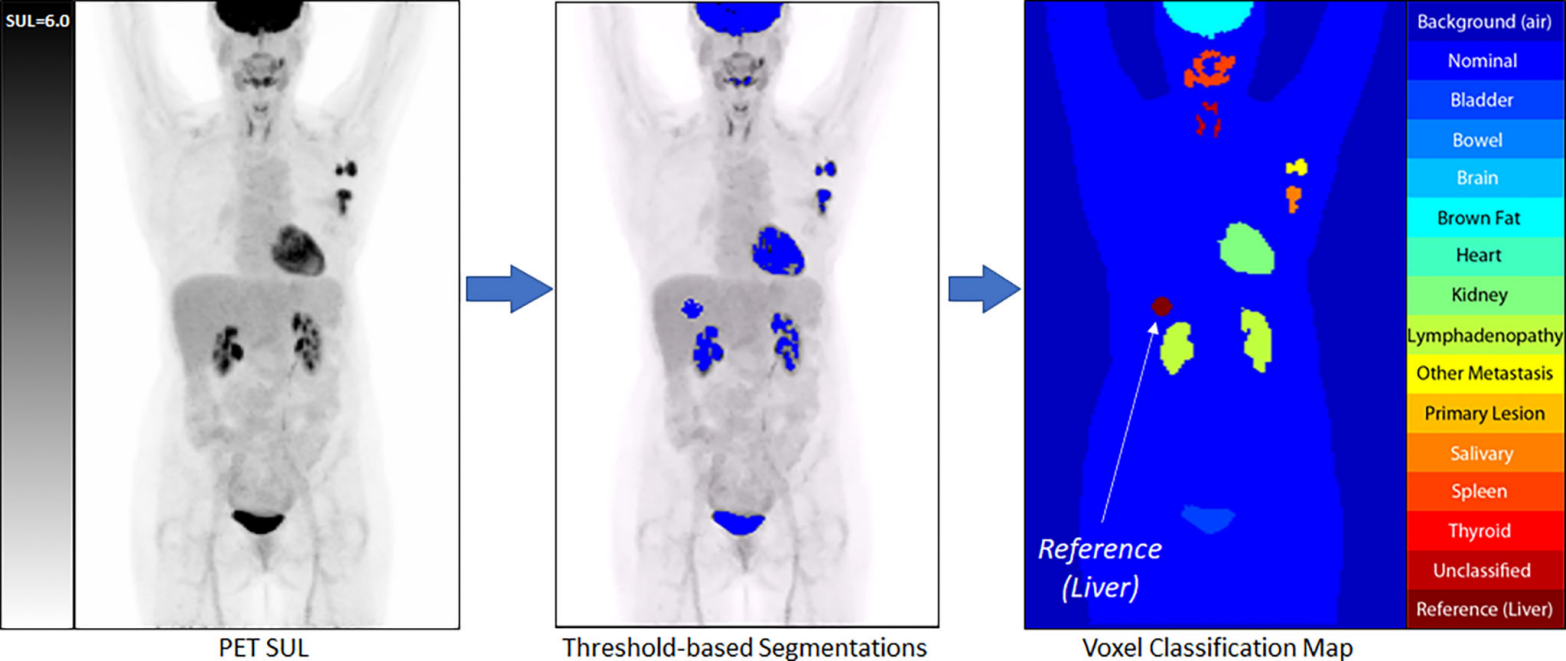
An example of our semi-automated threshold-based segmentation algorithm for the generation of training data.

All clusters of 7 or more connected voxels above that threshold were then automatically segmented and manually classified by a trained nuclear medicine physician to one of the tissue classes. A body contour was automatically generated using a partitioning threshold of 0.1 SUL. Voxels within the contour that were not otherwise classified were assigned the class of Nominal, with voxels outside the contour assigned the class Background. The auto-located 3 cm^3^ spherical Liver Volume of Interest (VOI) was assigned the class of Reference, which served as a proxy for the liver, although the liver’s avidity, by definition, was primarily below the threshold for segmentation [**Figure 2**] and, thus, not anatomically represented by the VOI.

With all PET voxels now assigned to a class, the matched PET, CT (re-sampled to match the PET images), and voxel classification images were exported as co-registered and voxel matched DICOM files. Each imaging study was then re-sampled in 3D to have isotropic voxels measuring 3.5 mm, and the transaxial image slices were center cropped to a final size of 128 x 128 voxels. These data were then re-processed using an automated data cleaning routine in which the PET component was filtered with a 3D spherical smoothing filter measuring 1 cm^3^ to reduce image noise. The Reference class voxels were then re-sampled, and a new reference mean and standard deviation were calculated. From these, a Reference Threshold was calculated.


$$\text{Reference Threshold}=1.0\times {\text{Mean}}_{\text{Liver}}-2.0\times \text{Std}.\text{ Dev}._{\text{Liver}}$$

  Equation 2 Reference Threshold

Using the Reference threshold, the Reference region was allowed to grow into the pool of adjacent voxels which were equal to or greater than the threshold and not otherwise classified (Nominal voxels only). After each iteration of Reference region growth, the pool of eligible voxels was commensurately ‘shrunk’ by 1 voxel, constraining the pool of voxels eligible for Reference growth during the next growth cycle. This technique, which we called ‘Round-Robin’ region growing, provided a self-limiting constraint to region growth and allowed the Reference region to grow into a more anatomically correct configuration while minimizing the opportunity for growth beyond the anatomic boundary.

Once Reference region growth self-terminated, a new Reference (Liver) mean and standard deviation was measured, and a new tissue threshold was calculated using the PERCIST Follow-up assessment threshold (19).


$$\text{Follow}-\text{up Assessment Threshold}=1.0\times {\text{Mean}}_{\text{Liver}}+2.0\times \text{Std}.\text{ Dev}._{\text{Liver}}$$

  Equation 3 Follow-Up Assessment Threshold

Using this new, slightly lower threshold than the one originally used, specific tissue classes were allowed to automatically grow into eligible (Nominal) adjacent voxels using the ‘Round-Robin’ approach used in the auto-growing of the Reference sample. As with the Reference region, the purpose of this second round of region growing was to allow the higher avidity-based segmentations to grow into the contour of their anatomy more closely. Once the annotation growing self-terminated, the corresponding PET, CT, and newly generated tissue annotations were exported for use in network training.

The PET data were exported in SUL units multiplied by 100 (e.g., a voxel with a value of 3.27 SUL was encoded in the training image as 327). The CT data were exported in Hounsfield (HU) units offset to a base value of ‘0’. Corresponding PET and CT transaxial slices were then concatenated into a single multi-channel 2D matrix and exported as a binary file for use in network training. The corresponding tissue annotations were exported as 8-bit indexed PNG format files. All files representing the same transaxial slice shared the same filename.

**Figure 3:**
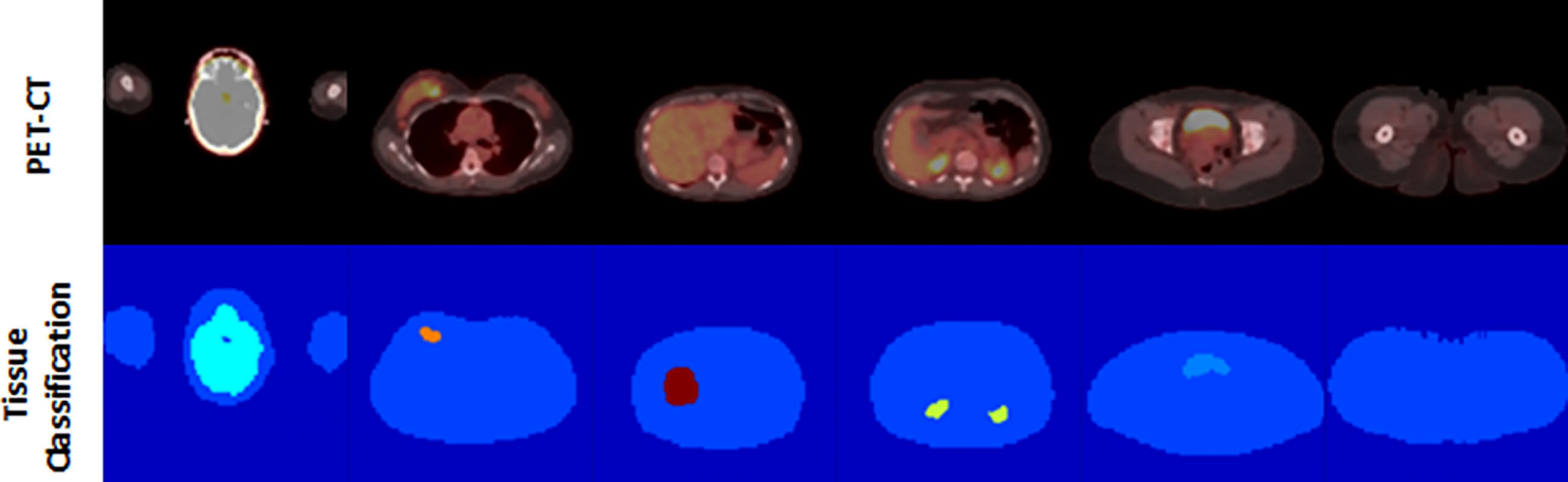
Example of PET/CT and tissue classification map pairings.

Of note, not every study contained voxels from every tissue class, as not every tissue (e.g., Brown_Fat) was present or expressed adequate avidity (e.g., Heart) in every study to be identified by the threshold-based method. In addition, the system was designed to process and learn from 2D transaxial plane images, so individual images used in the training could contain as few as two classes (Background and Nominal), or more [**Figure 3**].

The assignment of voxels to tissue classes was not uniformly distributed throughout the training set, with the number of voxels assigned to Background and Nominal far outweighing the number assigned to the segmented organs and lesions. To remedy this imbalance, class weights were calculated based on the total number of voxels in each class versus the entire training set, and their reciprocal values applied as weights during training. These weights were then use by the loss function of network when performing final pixel classification.

### Neural network architecture

**Figure 4:**
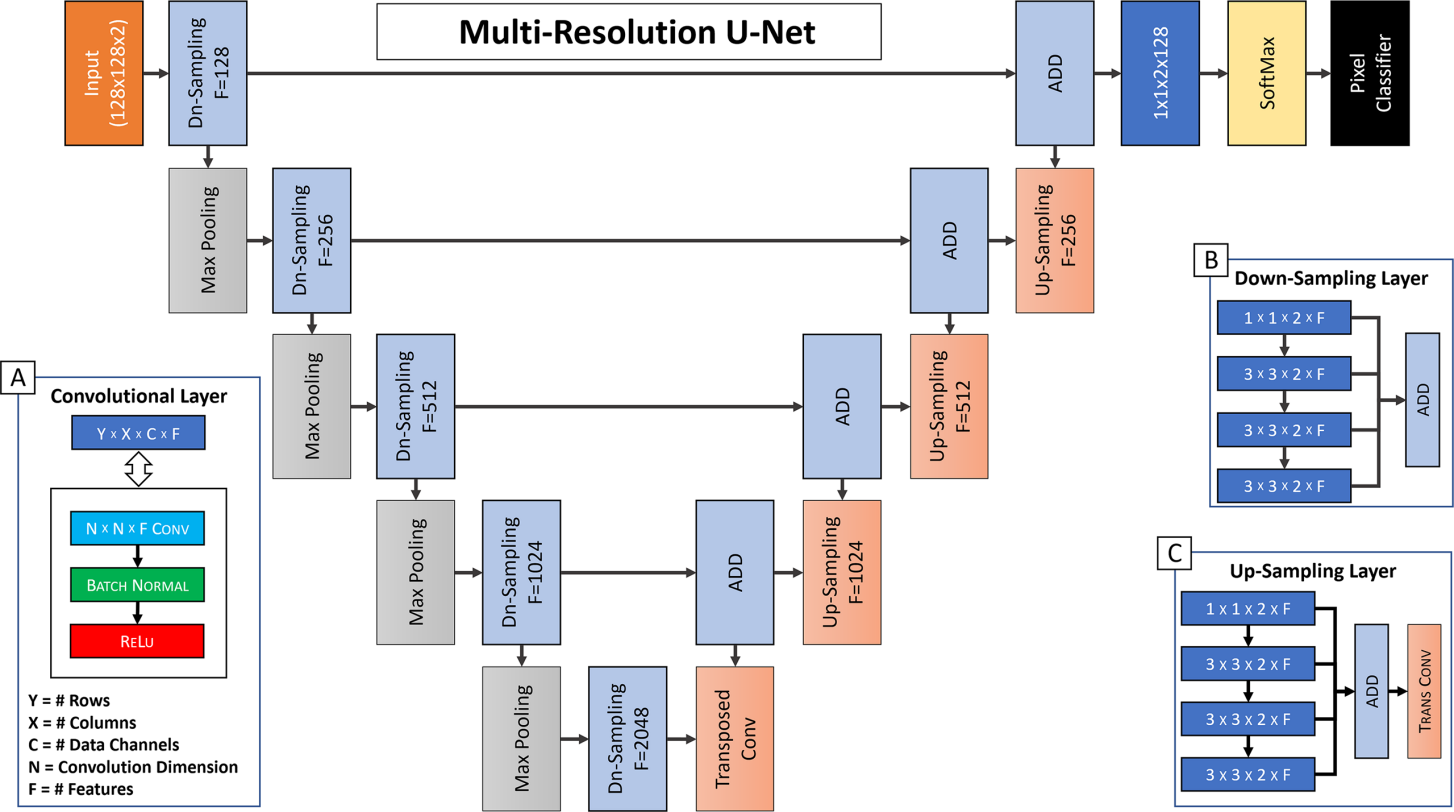
Our CNN Multi-Resolution Architecture, with details of the core Convolutional Layer **(A)**, the Down-Sampling Layer **(B)**. and the Up-Sampling Layer **(C)** Multi-Resolution Compound Structures.

Our CNN architecture [**Figure 4**] was modelled after a 2-dimensional (transaxial slice-based) multi-resolution U-Net (20). The network utilized 4 down-sampling layers [**Figure 4B**], and 4 up-sampling layers [**Figure 4C**]. The input layer had spatial dimension of 128 x 128 and a channel depth of 2, incorporating the PET and CT slices. The initial feature set size was 128. Each subsequent layer was reduced in spatial dimension by a factor of 2, while its feature size was increased by a factor of 2, until a bottleneck layer of size 8 x 8 x 2 x 2048 was achieved. We followed each convolutional layer with a pair of batch normalization and ReLu activation layers [**Figure 4A**]. The last layers of the network used a 1 x 1 convolutional layer with a reduction to 16 features, corresponding to our 16 tissue classes. This was followed by a batch normalization, SoftMax, and final pixel classification layer which used a class-weighted cross-entropy loss function.

### Network training strategy

As previously described, a total of 130 baseline PET/CT imaging studies were utilized. A study-level K-Folds (K=5) validation strategy was followed for training, validation, and testing of our network architecture. This resulted in five separate training/validation/testing sets composed of 104/13/13 studies each. Using a study-level strategy insured that there was no mixing of same patient image slices between training/validation/testing data sets.

Network training utilized a stochastic gradient descent with momentum (SGDM) solver as well as image augmentation, where in-plane image translation of ± 5 pixels, in-plane image rotation of ± 10 degrees, and image scaling between 50% to 200% of the original size were each independently and randomly employed for each image during each training epoch.

### Post-processing

The raw results obtained running the test data through the trained CNN were processed in a fully automated manner, not unlike the fully automated processing of the training data. The AI-generated annotations were first reconstructed in 3D. The Reference annotations were projected onto the PET image data and the mean and standard deviation were measured. A threshold was calculated using the follow-up assessment formula [Equation 3], and each tissue annotation was then cleared of voxels that were not equal to or greater than this threshold, with those voxels reassigned to the Nominal class. VOIs of the remaining annotated voxels were generated, and quantitative analysis of the resulting structures was performed.

### Quantitative analysis

The performance of our workflow was assessed at both the network level as well as the individual study level. At the network level, we calculated the confusion matrices for each K-Fold run. With the Lymphadenopathy class occurring in the training set at an average frequency of one-fifteenth the rate of the Primary_Lesion class, and both classes representing disease, we calculated the confusion matrices with the Primary_Lesion and Lymphadenopathy pooled into a single class, henceforth referred to as the Disease class.

At the study level, we calculated performance metrics for the automated detection of the aggregate Disease class. Metrics include Dice and Jaccard scores (measures of similarity to the ground truth), as well as a comparison of the measured SUL-Max.

## Results

### Network-level evaluation

Our network-level evaluation calculated performance metrics at the voxel level. The confusion matrices for each K-Fold session were assembled, with the True Positive (TP), False Positive (FP), True Negative (TN), and False Negative (FN) voxel classifications counted, and global performance metrics for each K-Fold calculated. This was performed on the raw CNN results, the raw auto-post-processed annotations, and again after pooling the Primary_Lesion and Lymphadenopathy classes into a single Disease class. Focusing on the post-processed results using the pooled Disease class, we calculated the mean values across the 5 K-Folds for the Sensitivity, Specificity, DICE, and Jaccard scores [**Table 2**] for each tissue class.

**Table 2:** Tissue specific performance metrics of the workflow in detecting and segmenting disease lesions in test studies averaged over all K-folds.

Tissue Class	Sensitivity	Specificity	DICE	Jaccard
BACKGROUND	0.99	0.98	0.99	0.98
NOMINAL	0.98	0.99	0.98	0.96
BLADDER	0.96	1.00	0.85	0.74
BOWEL	0.78	1.00	0.59	0.42
BRAIN	0.99	1.00	0.92	0.85
BROWN_FAT	0.78	1.00	0.32	0.20
HEART	0.96	1.00	0.95	0.90
KIDNEY	0.94	1.00	0.88	0.79
OTHER_METASTASIS	0.60	1.00	0.14	0.08
***PRIMARY_LESION***	***0.96***	***1.00***	***0.94***	***0.89***
SALIVARY	0.97	1.00	0.66	0.50
SPLEEN	0.63	1.00	0.20	0.12
THYROID	0.81	1.00	0.61	0.45
NOT_SIGNIFICANT	0.83	1.00	0.12	0.07
REFERENCE	1.00	1.00	1.00	1.00

The bold and italicized data are the results of the Disease class, which is the primary focus of our work.

### Study level evaluation

**Figure 5:**
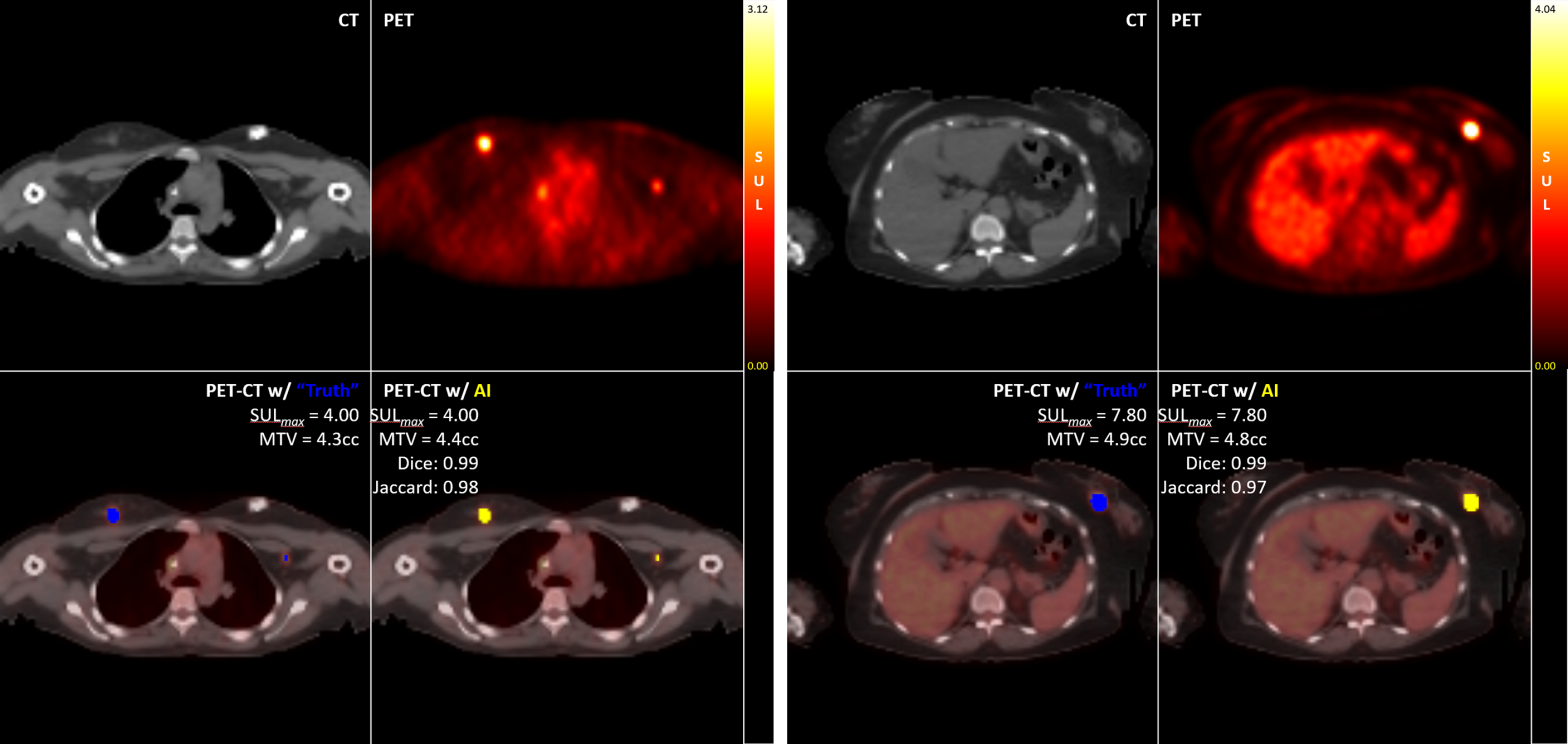
Examples of “Truth” vs “AI” detections and quantification of disease in two test cases.

For the study-wise evaluation, each PET/CT study in the test set was separately analyzed for 3D DICE, Jaccard scores, and percent of difference in SUL-Max (%ΔSUL↑) for the Disease class only using the auto-post-processed results across each K-Fold. Representative examples illustrating “Truth” vs “AI” detections are presented here [**Figure 5**].

Of the 65 total test cases (13 cases across 5 K-Folds), 4 cases failed to detect any Disease, which concurred with the case specific Ground Truth (no disease identified at the study-specific threshold). In addition, 2 other cases detected disease where Ground Truth failed. These 6 cases were independently reviewed by experienced nuclear medicine physicians, and the AI results were determined to accurately reflect the expected outcome of a typical, manually performed clinical review. For our analysis, we first calculated the DICE, Jaccard, and %ΔSUL↑ performance metrics by removing the studies with incalculable scores (‘CLEANED’) [**Table 3**], then again by replacing the incalculable scores with perfect scores for that metric (a value of ‘1.0’ for DICE and Jaccard scores, a value of ‘0%’ for the %ΔSUL↑) (‘CORRECTED’) [**Table 4**].

**Table 3:** Performance metrics for test data with non-scorable studies removed.

CLEANED Test Data			
N=59	DICE	JACCARD	%Δ ↑SUL
***Average***	0.91	0.85	0%
***St. Dev***	0.12	0.15	5%
***Median***	0.93	0.88	0%

**Table 4:** Performance metrics for test data with non-scorable studies assigned perfect scores.

CORRECTED Test Data			
N=65	DICE	JACCARD	%Δ ↑SUL
***Average***	0.92	0.86	0%
***St. Dev***	0.11	0.15	5%
***Median***	0.95	0.91	0%

## Discussion

Artificial intelligence is increasingly playing a significant role in image analysis within the field of radiology (1). Many are building systems that can assist and augment radiologic interpretations (2). [^18^F]FDG PET/CT plays a significant role in detection and management of a variety of oncologic abnormalities (21). Breast cancer is often FDG-avid on PET/CT and is a leading cause of cancer and cancer-related mortality in women (22). While studies have shown that [^18^F]FDG PET/CT is not effective in the evaluation of local disease, it plays a significant role in the management of patients with locally advanced disease and inflammatory carcinoma (23). Patients with clinical stage IIB disease (T2N1/T3N0) or higher may also benefit from evaluation by [^18^F]FDG PET/CT (24). Finally, quantification by [^18^F]FDG uptake on PET/CT after initiating therapy may identify responders from non-responders early, allowing new therapies to be pursued for those non-responders (15, 19). AI-augmented analysis of PET examinations may, in addition to aiding radiologic detection and therapeutic monitoring, provide additional data that may not be discernable from qualitative analysis but that can direct therapeutic regimens for individual patients.

As evidenced by the results, our AI-based framework had a high rate of voxel-wise accuracy at classifying most FDG avidity. In the evaluation of the test data, conducted by trained readers, it demonstrated very high sensitivity and specificity at identifying avidity associated with breast cancer.

The performance of this framework was achieved utilizing traditional, standards-based image processing in concert with the DL-CNN. The combination of these methods, along with a heterogeneous training dataset, achieved a level of automated performance that neither method on its own could, especially considering the relatively low number of studies and samples per class used (25) (26). The PERCIST v1.0 framework provided an objective methodology for the generation of training data as well as the post-processing refinement of the AI-generated classifications. The DL-CNN provided the classification key that was necessary for automated analysis.

## Conclusion

This pilot work has demonstrated the ability of an AI-based workflow, incorporating standards-based data processing paired with DL-CNNs, to specifically identify malignant breast tissue as demonstrated by [^18^F]FDG avidity in a PET/CT study. This supports the idea that AI can be trained to recognize specific tissue signatures in molecular imaging studies using radiopharmaceuticals. Future work will explore the applicability of these techniques to other disease types and alternative radiotracers, as well as explore the accuracy of fully automated quantitative analysis and response assessment.

## Data availability statement

The data analyzed in this study is subject to the following licenses/restrictions: Access to datasets for this study will be considered for serious researchers upon reasonable request. Requests to access these datasets should be directed to jleal1@jhmi.edu.

## Ethics statement

The studies involving human participants were reviewed and approved by Johns Hopkins Institutional Review Board. The patients/participants provided their written informed consent to participate in this study.

## Author contributions

JL, SR, MP, and LS contributed to conception and design of the study. JL and LS performed the analysis. JL, VS, RC, CV, ML, AS, and RW collected the data. JL wrote the first draft of the manuscript. SR and LS wrote sections of the manuscript. All authors contributed to the article and approved the submitted version.

## Funding

The authors wish to acknowledge the following for their support of this work: National Institutes of Health/National Cancer Institute P30CA006973, the National Institutes of Health/National Cancer Institute U01-CA140204, the Translational Breast Cancer Research Consortium, the funding support to the TBCRC from the AVON Foundation, The Breast Cancer Research Foundation, and Susan G. Komen, and the Nvidia Corporation GPU Grant Program.

## Acknowledgments

The authors wish to acknowledge the following for their support of this work: National Institutes of Health/National Cancer Institute P30CA006973 the National Institutes of Health/National Cancer Institute U01-CA140204, the Translational Breast Cancer Research Consortium, the funding support to the TBCRC from the AVON Foundation, The Breast Cancer Research Foundation, and Susan G. Komen, and the Nvidia Corporation GPU Grant Program.

## Conflict of interest

JL and SR are consultants of PlenaryAI. MP is a co-founder of PlenaryAI.

The remaining authors declare that the research was conducted in the absence of any commercial or financial relationships that could be construed as a potential conflict of interest.

## Publisher’s note

All claims expressed in this article are solely those of the authors and do not necessarily represent those of their affiliated organizations, or those of the publisher, the editors and the reviewers. Any product that may be evaluated in this article, or claim that may be made by its manufacturer, is not guaranteed or endorsed by the publisher.
